# Indoor Location Sensing with Invariant Wi-Fi Received Signal Strength Fingerprinting

**DOI:** 10.3390/s16111898

**Published:** 2016-11-11

**Authors:** Mohd Nizam Husen, Sukhan Lee

**Affiliations:** Intelligent Systems Research Institute, Sungkyunkwan University, Suwon, Gyeonggi-do 440-746, Korea; mnizam@skku.edu

**Keywords:** indoor positioning, Wi-Fi fingerprinting, WLAN indoor localization, smartphone sensor, received signal strength (RSS)

## Abstract

A method of location fingerprinting based on the Wi-Fi received signal strength (RSS) in an indoor environment is presented. The method aims to overcome the RSS instability due to varying channel disturbances in time by introducing the concept of invariant RSS statistics. The invariant RSS statistics represent here the RSS distributions collected at individual calibration locations under minimal random spatiotemporal disturbances in time. The invariant RSS statistics thus collected serve as the reference pattern classes for fingerprinting. Fingerprinting is carried out at an unknown location by identifying the reference pattern class that maximally supports the spontaneous RSS sensed from individual Wi-Fi sources. A design guideline is also presented as a rule of thumb for estimating the number of Wi-Fi signal sources required to be available for any given number of calibration locations under a certain level of random spatiotemporal disturbances. Experimental results show that the proposed method not only provides 17% higher success rate than conventional ones but also removes the need for recalibration. Furthermore, the resolution is shown finer by 40% with the execution time more than an order of magnitude faster than the conventional methods. These results are also backed up by theoretical analysis.

## 1. Introduction

Indoor location-based services (LBS) have attracted much attention due to their commercial and social values. Applications of these services in the fields of robotics, logistics, medicine, tourism, and entertainment are expected to grow in the coming years. A variety of sensor technologies could be utilized as a medium to determine indoor location. Radio Frequency (RF) signals such as ultrasound [1], Bluetooth [2], radio frequency identification (RFID) [3], infrared [4], ultra-wide band (UWB) [5], ZigBee [6], and Wi-Fi [7] have been used in the past and present of LBS and indoor localization work. Among those mentioned, Wi-Fi signals using received signal strength (RSS) fingerprinting has been considered as one of the most popular indoor positioning solutions due to its low cost [8]. Wi-Fi RSS fingerprinting technique consists of two phases: offline site survey and online pattern matching. During the offline phase, the RSS values of several Wi-Fi access points are collected at different predefined calibration locations to create a fingerprint database named radio map. Then, during the online phase, RSS samples are collected in real-time and matched with RSS patterns pre-stored in radio map to estimate the user’s location.

The utilization of Wi-Fi RSS for indoor location fingerprinting has drawn attention as an enabler of various location-based personal services with handheld and wearable communication devices. However, the instability of Wi-Fi RSS incurred by highly mutable channel characteristics, due to random spatiotemporal disturbances, hampers a wide-spread adoption of RSS-based location fingerprinting to real world applications [8,9]. Conventional methods adopt the multi-class pattern classification framework for location fingerprinting, where the RSS sensed from available Wi-Fi sources is collected at each calibration location as statistical samples to form a pattern class. Fingerprinting is then performed by deriving the decision boundaries that provide minimum-error classification [10]. The problem is that the random spatiotemporal disturbances associated with RSS make class patterns heavily overlapped and time varying, causing significant classification errors while requiring frequent offline recalibration. Increasing the number of available Wi-Fi sources and/or decreasing the number of calibration locations may help reduce the problem somewhat, but at the expense of efficiency and/or fingerprinting resolution. Although Wi-Fi sources ineffective for classification can be identified and removed offline for improved efficiency [11], the problem described above remains fundamentally unchanged.

This paper presents a novel method to cope with the RSS instability, using the concept of “the minimally disturbed invariant RSS statistics”, or, simply, “the invariant RSS statistics”. The invariant RSS statistics introduced here are intended not only for enhancing the performance of classification but also for eliminating the need for offline recalibration without reducing fingerprinting resolution and efficiency. The main purpose is to determine a real-time location of a Wi-Fi sensor device based on the real-time RSS values, evaluating these against the database for the closest pre-defined calibration locations. As such, the proposed method is especially suited for smartphone-based indoor location fingerprinting where only the Wi-Fi sources existing in the environment are to be utilized. We conducted an experimental study and theoretical analysis to support the results. The contributions of this article are summarized below:

1. A new method is introduced by resorting to invariant RSS statistics as the reference in fingerprinting, together with the effective RSS readings chosen as input data, which make the proposed fingerprinting accurate and robust against random spatiotemporal disturbances.
2. The automatic removal of ineffective Wi-Fi signal sources in the process of soliciting effective RSS readings makes the proposed method efficient in fingerprinting with the in-situ reduction in the dimensions of decision space.
3. A proposed design guideline is presented as a rule of thumb for estimating the number of Wi-Fi signal sources required to be available for any given number of calibration locations under a certain level of random spatiotemporal disturbances. This will serve as a key guideline for the benefits of the society who wish to employ invariant RSS-based indoor localization using Wi-Fi fingerprinting.
4. Our method requires no recalibration once the invariant RSS statistics are set initially, unlike conventional methods which require recalibration after a certain period of time. This contributes better localization success rate with stable performance in time.

The rest of the paper is organized as follows: in Section 2, we review existing works in indoor localization and Wi-Fi fingerprinting. Section 3 elaborated thoroughly our proposed method and design guideline. Consequently, the experimental results are presented in Section 4. Then, in Section 5 we present the theoretical analysis to support our experimental results. Finally, Section 6 concludes the paper.

## 2. Related Work

In radio frequency-based indoor positioning, the RADAR system of Microsoft Research [10] was the first work that made use of the WiFi-based network to produce location fingerprints. In the training phase, an area is divided into a 1 × 1 m grid where the signal strength measurements of the access points are taken at each intersection. The mean of the signal strengths which have been obtained is recorded to create a radio map to be used in the online phase. In the online phase, when the user looks for its location, the mobile station will detect and record the signal strength from as many access points as possible. Then, the signal strength received will be compared to the radio maps to determine the location of the user. Although the authors assume that the radio map is always stable, there is an issue in that this is not always the case [8]. Furthermore, by picking the access points’ signal strength arbitrarily, it is hard to get the optimum location estimation.

In indoor localization, there are two methods for identifying the location: the deterministic and the probabilistic method [12]. Both methods have its own strengths. Bolliger [13] explored the issue of localization accuracy using multiple deterministic and probabilistic methods based on WLAN fingerprinting to improve the radio map precision in the database. Bolliger has developed an indoor positioning system named Redpin in the implementation of their approach. There is also a method using signal strength ratios between pairs of base stations [14]. They claim that this method is more stable among Wi-Fi clients than absolute signal strength. This approach, however, will increase the computational time. There are also other similar works [15,16] trying to use Wi-Fi RSS in the fingerprinting process.

Recently, research in WiFi-based indoor localization explored its feasibility using smartphones with various techniques [11,17,18,19,20,21,22,23,24,25,26,27,28]. In choosing the Wi-Fi signals to be used to uniquely classify a location, different authors used different approaches in their methods. However, none of them have yet successfully created a stable location fingerprint database because of the nature of Wi-Fi signals that fluctuates over time depending on environment disturbance.

Martin et al. [20] claim that they are the first to implement localization application using RSS of Wi-Fi signals in a smartphone. The authors apply the deterministic method in their work. They take all signals above −80 dBm and used their mean values in their location fingerprint database formation. A similar approach is also implemented by Shin et al. [21]. They developed an indoor Wi-Fi positioning system for Android-based smartphone and collecting Wi-Fi access points RSS indiscriminately. By this approach, the localization process is suffering from a good accuracy because of instability to the location fingerprints due to signal fluctuations.

Meanwhile, Chen [22] use Received Signal Strength (RSS) relations instead of RSS mean values. They claim that although RSS fluctuates, relations between values are more stable. Their approach is setting up rules at each reference location such as “*less than*”, “*equal to*”, or “*greater than*” between two related signals. Another method [24] proposed a way to improve location estimation by penalizes signals from unstable access points and signifies strong signals compared to weak signals. They criticized the use of Euclidean distance in nearest neighbor approach. However, all these approaches still will lead to the same problem where variation is too high when time varying is included during data collection, which will lead to unavoidable large overlaps in location classification.

Another approach in the selection of Wi-Fi signals is using a *predetermined number* of “first-come-first-get” signals [25]. By setting up a predetermined number (e.g., 8), they will compare the online measured first eight detected signals to the location fingerprint database to estimate the location. The authors apply the probabilistic method in this work. Although this approach will slightly increase the computational time, again it will suffer from instability of accuracy. The work in [19] focusing on orientation-based indoor localization using smartphones, where they managed to localize the smartphone by location and orientation. However, there is still rooms for improvements in the calibration phase to avoid inaccurate detection.

The key to formulating a stable location fingerprint is selecting the best appropriate signals. Samih Eisa et al. [11] has made an effort in this matter by removing useless signals. However, their approach still lacks in producing highly stable location fingerprints because the author’s definition and identification methods of useless signals are not enough to select the best signals to form a good location fingerprints.

All the above mentioned past related researches have issues with random spatiotemporal disturbances and do not provide any design guideline to implement their approach. This paper proposes an accurate and robust Wi-Fi RSS fingerprinting against random spatiotemporal disturbances based on the concept of invariant RSS [28] and a design guideline that relates statistically among the number of Wi-Fi sources, the number of calibration locations, the level of disturbance, and the success rate.

## 3. Methodology

This section will elaborate the Wi-Fi received signal strength (RSS) model and the invariant RSS method, followed by the design guideline of implementing invariant RSS indoor localization.

### 3.1. Invariant Wi-Fi RSS Method

**Figure 1:**
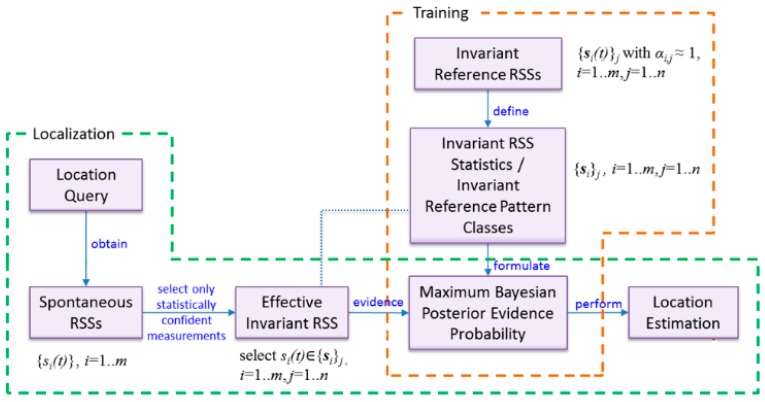
Proposed method of the smartphone-based indoor location fingerprinting based on invariant Wi-Fi received signal strength.

Figure 1 illustrates the overview of the processes in the proposed invariant RSS method for indoor localization using a smartphone. The proposed method consists of two phases. It starts with the training phase, where the essential *invariant* reference pattern classes are formed based on invariant RSS statistics. Then, the localization phase is carried out to estimate the user’s location based on real-time Wi-Fi RSS sensing.

The *m* dimensional RSS vector, {***s**_i_*(*t*)}*_j_*, at time *t* at a particular calibration location *j* due to *m* Wi-Fi sources, *i* = 1, …, *m*, is represented as:

(1) $${\left\{s_{i}\left(t\right)\right\}}_{j}=\left\{s_{1,j}\left(t\right),s_{2,j}\left(t\right),\;\ldots ,s_{i,j}\left(t\right),\ldots ,s_{m,j}\;\left(t\right)\right\},\;j=1,\ldots ,n$$

where the bold-face letter, ***s***, is used to represent it as a random variable.

Here, we model ***s**_i,j_*(*t*), the RSS from the *i*th Wi-Fi source at the *j*th calibration location, as:

(2) $$s_{i,j}\left(t\right)=\;\alpha_{i,j}\left(t\right)\times \;r_{i,j}+\delta_{i,j}$$

where *r_i,j_* represents the time-invariant RSS with no spatiotemporal disturbances present, ***α**_i,j_*(*t*) the multiplicative signal alteration factor to account for the spatiotemporal disturbances of *r_i,j_*, and ***δ**_i,j_* the sensor noise. Note that we introduce *r_i,j_* as the ideal time-invariant signal attenuated by the distance from the signal source *i* to the location *j* through the invariant channel characteristics, taking only fixed building infrastructure and furniture layout into consideration.

The actual signal, ***s**_i,j_*(*t*), is then considered as the alteration of *r_i,j_* by ***α**_i,j_*(*t*), 0 < ***α**_i,j_* ≤ 1, reflecting the stochastic channel characteristics due to randomly moving people and/or objects as well as randomly orienting smartphone users disturbing the channel during RSS measurement.

Let us define the invariant RSS, ***ŝ**_i,j_*(*t*), as ***s**_i,j_*(*t*) with minimal random spatiotemporal disturbances, i.e., ***α**_i,j_* ≈ 1. Then, from Equation (2):

***ŝ**_i,j_*(*t*) ≈ *r_i,j_* + ***δ**_i,j_* (3)

**Figure 2:**
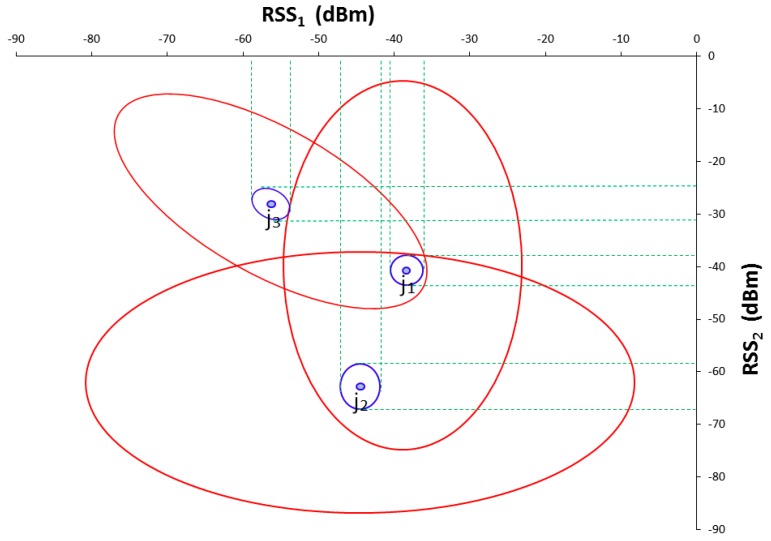
Schematic representation of three reference pattern classes in 2-dimensional Wi-Fi source space that illustrates the difference between the distribution of invariant reference pattern classes (small blue ellipses) and the spatiotemporal varying RSSs (large red ellipses).

Since the randomness of ***ŝ**_i,j_*(*t*) comes mostly from sensor noise, ***δ**_i,j_*, multiple measurements of ***ŝ**_i,j_*(*t*) form a cluster around *r_i,j_*, the statistics of which is governed by that of ***δ**_i,j_*. Assuming that the statistics of ***δ**_i,j_* are time-invariant, so is ***ŝ**_i,j_*(*t*). For convenience, the statistics of ***ŝ**_i,j_*(*t*) are represented as a random variable, ***s**_i,j_*. Then, at the calibration location *j* with *m* available Wi-Fi sources, an *m* dimensional vector of invariant RSS statistics, {***s**_i_*}*_j_*, *i* = 1, ..., *m*, is defined. For the pattern classification point of view, {***s**_i_*}*_j_* represents the *j*th reference pattern class in the *m* dimensional space of Wi-Fi sources. The small blue ellipses in Figure 2 schematically represent three invariant reference pattern classes, {***s**_i_*}*_j_*, *j* = 1, 2, 3, in two-dimensional Wi-Fi source space *i* = 1, 2. Notice that the red ellipses surrounding the blue ellipses represent spatiotemporally varying RSS statistics.

Fingerprinting of the RSS vector, {*s_i_*(*t*)}, captured at an unknown location is done by identifying the reference pattern class among {***s**_i_*}*_j_*, *j* = 1, …, *n*, that maximally support {*s_i_*(*t*)}. The support of {*s_i_*(*t*)} by the reference pattern class *j* is defined here as the number of *s_i_*(*t*) that fall in ***s**_i,j_* for *i* = 1, …, *m*, or the sum of the likelihood probabilities of *s_i_*(*t*) to belong to ***s**_i,j_*, for *i* = 1,…, *m*. Note that, in counting the number or summing the likelihood probabilities, only those *s_i_*(*t*) having a sufficiently high level of statistical confidence as a member of ***s**_i,j_* are allowed to participate in the sum, while excluding others. That is, *s_i_*(*t*) ∈ ***s**_i,j_*, for *i* = 1, …, *m*, when the following equation is satisfied:

(4) $$\left|s_{i}\left(t\right)-\mu \left(s_{i,j}\right)\right|\;<\;\xi \sigma \left(s_{i,j}\right)$$

where μ(***s**_i,j_*) and σ(***s**_i,j_*) are the mean and the standard deviation of ***s**_i,j_*.

Fingerprinting is done based on the pattern classification rule as shown in Algorithm 1. Notice that, in the algorithm, when two or more calibration locations come up with the same support values, the decision is delayed and signals are recaptured until only a single calibration location produces the maximum support.
**Algorithm 1.** Pseudocode of the localization phase in estimating the user’s location based on real-time spontaneous sensed Wi-Fi RSS vector.**Input:** Spontaneous RSS Vector Measurement {*s_i_*(*t*)} **Output:** Estimated Location *L*∈{*1,..,n*}1: *m* ← Number of Wi-Fi Sources2: *n* ← Number of Calibration Locations3: ***s**_i,j_* ← A component of *{**s**_i_}_j_*, representing Invariant RSS Pattern for the *i*th Wi-Fi  source at Calibration Location *j*4: σ(***s**_i,j_*) ← Standard Deviation of ***s**_i,j_*5: ξσ(***s**_i,j_*) ← Decision Margin6: **for**
*j* = 1 to *n*
**do**7:  **for**
*i* = 1 to *m*
**do**8:   **if**
*s_i_*(*t*) ∈ ***s**_i,j_*, i.e., |*s_i_*(*t*) *-* Mean of ***s**_i,j_* | < ξσ(***s**_i,j_*) **then**9:     Either *sum*(*j*) ← *sum*(*j*) + 1      or *sum*(*j*) ← *sum*(*j*) + Pr(*s_i_*(*t*)*| **s**_i,j_*)10:   **end if**11:  **end for**12:  **if**
*sum*(*j*) *> Maximum* (default: *Maximum* = 0.0) **then**13:   *Maximum = sum*(*j*)14:   *L* ← *j*15:  **else if**
*sum*(*j*)*= Maximum **then***16:   *L* ← 017:  **end if**18: **end for**19: **if**
*L = 0*
**then**20:  Reject and Recapture Signals21: **end if**

The proposed method provides better performance over conventional ones because we use the concept of invariant RSS statistics in the formulation of invariant reference pattern classes which produce low variance in avoiding RSS instability. Furthermore, during the localization phase, only the invariant RSS, i.e., the *s_i_*(*t*) having a sufficiently high level of statistical confidence as a member of ***s**_i,j_*, are selected to participate in classification in comparison to the conventional methods that takes all available RSS to participate in the classification. This will cause the proposed method deliver better performance with the low variance, thus, preventing RSS instability effects as encountered by the conventional methods. The other advantage of our proposed method is that by a proper choice of decision margin, a minimal false negative can be achieved with the expense of rejections. The slight rise in rejections is handled by repeating location queries which ultimately produces true positives. Experiments demonstrating the superior performance of our proposed method are detailed in Section 4.

### 3.2. Design Guideline of Invariant RSS Based Wi-Fi Fingerprinting

This section describes the formulation of a design guideline for the benefits of the society who wish to employ invariant RSS-based indoor localization using Wi-Fi fingerprinting in any indoor environment. The guideline represents how the relations between the numbers of Wi-Fi signals obtained with a certain number of calibration locations may contribute to the class/location separation degree that will classify and separate each calibrated location, at many different levels of random spatiotemporal disturbances. This useful guideline is produced based on statistical analysis. In formulating the design guideline, we develop a simulation algorithm. The simulation is developed in order for us to control the level of random spatiotemporal disturbance and the number of Wi-Fi sources. In real life, the spatiotemporal disturbance is random and difficult to set and control, while the obtainable number of Wi-Fi sources is depending on the surrounding environment. Hence, simulation is the best solution in order to formulate the design guideline.

#### 3.2.1. Design Guideline Development Procedure

**Figure 3:**
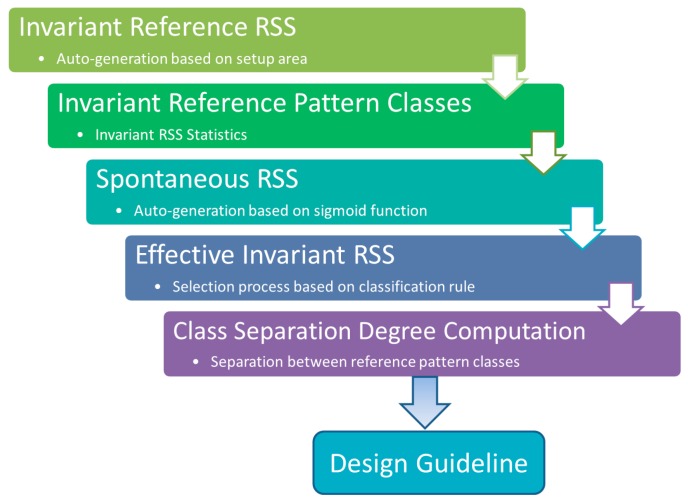
Procedure to formulate design guideline.

Figure 3 depicts the steps that have been carried out in formulating the design guideline. In the procedure, we simulate the invariant reference RSS propagations, spontaneous RSS propagations, identify the effective invariant RSS after applying spatiotemporal disturbances, and compute the class separation degree of the calibrated reference locations.

● **Step 1: Invariant Reference RSS Generation**

In collecting the invariant reference RSSs at each calibration location *j*, invariant reference RSS is defined by {***s**_i_*(*t*)}*_j_* with ***α**_i,j_* ≈ 1. We generate the signal propagation values based on the ***ŝ**_i,j_*(*t*) as defined in Equation (3). The value of ***ŝ**_i,j_*(*t*) is based on *r_i,j_*, where *r_i,j_* is the ideal time-invariant signal attenuated by the distance from the Wi-Fi signal source *i* to the location *j* through the invariant channel characteristics, taking only fixed building infrastructure and furniture layout into consideration. In the invariant RSS values generation, we set the following number of Wi-Fi signal sources *m* and the number of calibration locations *n* as defined in Table 1 to the same setup area.

**Figure 4:**
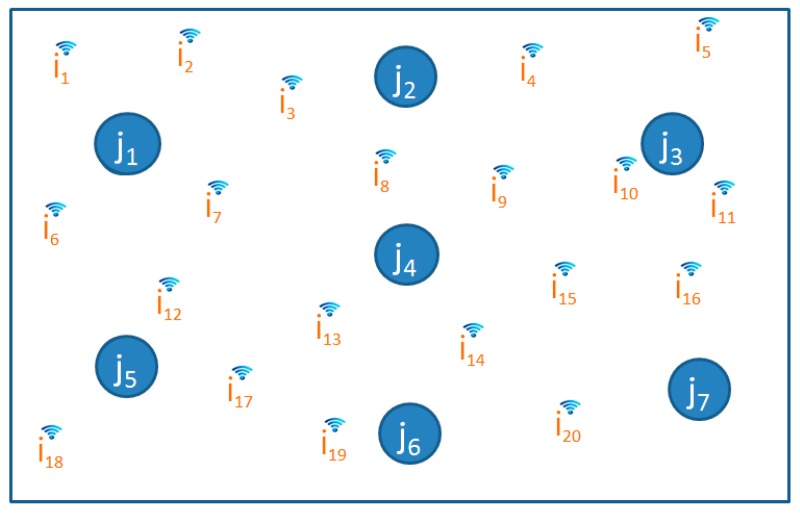
The visual representation of setup area to generate invariant reference RSS with 20 Wi-Fi signal sources *i* = 1, 2, …, *m*; and seven calibration locations *j* = 1, 2, …, *n*; (*m* = 20, *n* = 7).

As an example, when we simulate the area with seven calibration locations and 20 Wi-Fi sources as illustrated in Figure 4, we generate all 140 invariant reference RSSs. To get a statistical datapoint, we generate the reference RSS values repeatedly for 300 times. Then, the *mean* and *standard deviation* of each RSS at each calibrated location are calculated.

● **Step 2: Invariant Reference Pattern Classes Formulation**

After the invariant reference RSS are collected, the invariant RSS statistics, {***s**_i_*}*_j_*, as mentioned in Figure 1, are set and the invariant reference pattern classes are defined. At each calibration location *j*, with all available Wi-Fi signal sources, *i* = 1, 2, ..., *m*, will produce an *m* dimensional invariant RSS statistics vector, {***s**_i_*}*_j_*. From the invariant RSS statistics, the reference pattern classes are formulated and stored in a database.

● **Step 3: Spontaneous RSS Generation**

In order to simulate the spontaneous RSS gained at an unknown location during location query in a real-life situation, we developed another simulation module to act out the situation. The spontaneous RSS is the signals added by temporally varying signal disturbance, *α_i,j_*, due to the moving people and objects that may disrupt the channel and/or the orientation of the smartphone user disturbing or blocking the channel at the time of Wi-Fi sensor measure the RSS. The effect of *α_i,j_* on spontaneous RSS is represented by a multiplication coefficient to invariant reference RSS by executing Equation (2). The explanation of each parameter in the equation are already mentioned in Section 3.1. The level of α*_i,j_* values is selected in 0.1 intervals. At each level of *α_i,j_*, the associated α to each Wi-Fi source is distributed in the uniform random distribution of sigmoid function as:

(5) $$f_{1}\left(\alpha \right)=\frac{1}{1+e^{-\alpha }},\;0<\alpha <1$$

The reason to apply sigmoid function on α rather than just binary values or exponential function is to reflect the real life behavior of the disturbance towards Wi-Fi signals. Based on the experimental data analysis, it is observed that the effect of random spatiotemporal disturbance over RSS is modeled closest to sigmoid function in comparison to the others.

A sigmoid function values based on the number of Wi-Fi signal sources *m*, the number of calibration locations *n*, and the temporally varying signal disturbance level *α* as:

(6) $$f_{2}\left(\alpha \right)=\;f_{1}\left(\alpha \right)mn\;=\frac{1}{1+e^{-\alpha }}mn,\;0<\alpha <1$$

Then, we generate uniform random numbers and assign the numbers from left to the right of the sigmoid curve values. This represents the varying disturbances for each Wi-Fi RSS. Finally, the invariant reference RSS values of each Wi-Fi sources which already gained from the previous process is multiplied to the respective temporally varying signal disturbance by executing Equation (2). The outcome of the computation is the spontaneous RSS gathered at each calibrated location.

● **Step 4: Effective Invariant RSS Selection**

The selection of the effective signals to be used in fingerprinting classification and the removal of the ineffectual signals to discriminate and uniquely identify each calibrated location is the key factor for the success of a Wi-Fi fingerprinting method. The process is based on the condition in line 8 of the pseudocode as depicted in Algorithm 1, where the effective invariant RSS is the individual spontaneous RSS within the decision margin. In the simulation, we defined the effective invariant RSS as the *α* values being uniform randomly selected in the range between 0.9 and 1.0. The threshold value of 0.9 is determined based on the analysis of experimental data. From the data analysis, it was observed that in classifying an RSS as invariant or not, 0.9 produces the best result in comparison to 0.8 and 0.7. Hence, in defining the invariant and non-invariant RSS from the number of Wi-Fi signal sources *m* and the number of calibration locations *n* is:

(7) $$g\left(m,n\right)\;=\{\begin{array}{c} 1,\;f_{2}\left(\alpha \right)\geq 0.9\\ 0,\;f_{2}\left(\alpha \right)<0.9 \end{array}$$

● **Step 5: Class Separation Degree Computation**

Class separation degree refers to the level of discrimination power between pattern classes. It is calculated as the following process:

(8) $$\frac{N}{\sum_{k=1}^{n-1}k}$$

where *N* is the number of calibration locations that could be discriminated or separated by the intensity of the effective invariant RSS, *n* is the number of calibration locations, and the denominator is the total number of combination pairs.

In calibration locations *n* = 7, there are 21 possible combination pairs. So, to compute the class separation degree, we compute how many combination pairs out of the 21 that are successfully separate the calibration locations. The same method is used in 15 and 20 calibration locations setup where there are 105 and 190 pairs, respectively.

**Figure 5:**
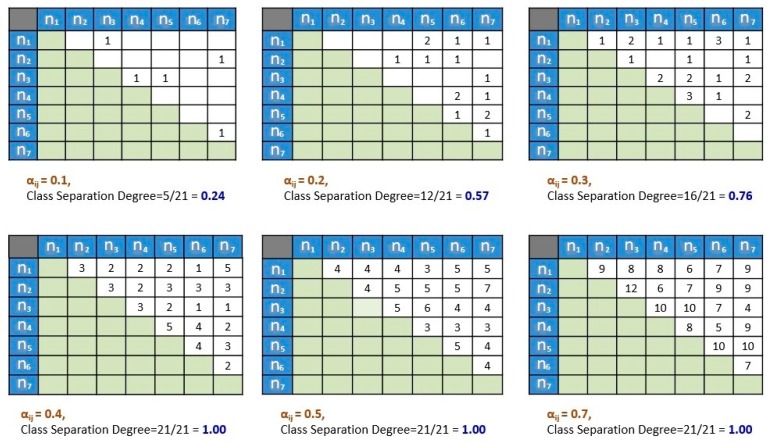
An example of the visualization in computing the class separation degree at (*n* = 7, *m* = 20). This example showing the computation of the class separation degree at different levels of disturbance: *α* = 0.1, 0.2, 0.3, 0.4, 0.5, and 0.7.

Figure 5 depicts the conception example of the class separation degree calculation process at *n* = 7, *m* = 20. There are 21 possible combinations pairs in seven calibration locations which represented by 21 (white) cells as shown in the figure. At a different level of random spatiotemporal disturbance *α*, the class separation degree is computed. The numbers presented in each cell represent the number of Wi-Fi signals that can discriminate the two intersects locations. Blank cell denotes no signals may discriminate both locations. Notice that the class separation degree starts to be 100% when α*_i,j_* = 0.4, and remains 100% when α*_i,j_* is incremented. Although it remains 100% as it is incremented, the numbers of Wi-Fi signals that can discriminate/separate each calibrated location are also getting higher. This shows that higher values of α*_i,j_* will give better location separation options.

#### 3.2.2. Proposed Design Guideline

**Figure 6:**
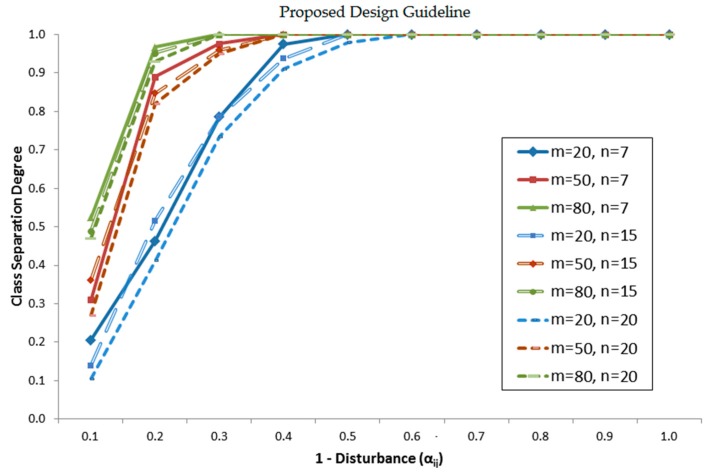
Design guideline for the number of Wi-Fi signal sources (*m*) and level of disturbance (*α*) for a given number of calibration locations (*n*) to achieve certain class separation degree.

After implementing the processes as mentioned in the previous subsection, we present the result as depicted in Figure 6 which significantly beneficial as a guideline to employ indoor localization using invariant RSS-based location fingerprinting. Based on this result, we conclude that the higher the number of Wi-Fi sources *m*, the higher the class separation degree. It means more obtainable signals at any particular location are better to uniquely identify that location. When the number of calibrated locations *n* is increased, it will lower the class separation degree as proved in the graph where the pattern shifted to the right. This is particularly happening at a lower number of Wi-Fi signal sources *m* = 20. The result also shows that at *m* = 80, the similar class separation degree is gained when *n* = 7, *n* = 15, and *n* = 20. That means when the accessible Wi-Fi sources *m* is 80, it is enough to discriminate all the maximum 20 calibration locations without deteriorating the class separation degree.

As a recommendation to employ indoor localization using invariant RSS-based location fingerprints, an optimum number of obtainable Wi-Fi signal sources is above 50 when the number of calibration location *n* is 20 to get a good class separation degree (above 90%). A lower number of *m* is tolerable as the number of *n* is gradually decreased.

## 4. Evaluation and Results

Prior to the theoretical investigation on the implication of the proposed method, an experimental evaluation of the performance of proposed method is conducted in comparison with such well-known conventional methods as *RADAR* [10] and *Removal of Useless Wi-Fi Sources* [11].

### 4.1. Experimental Setup

**Figure 7:**
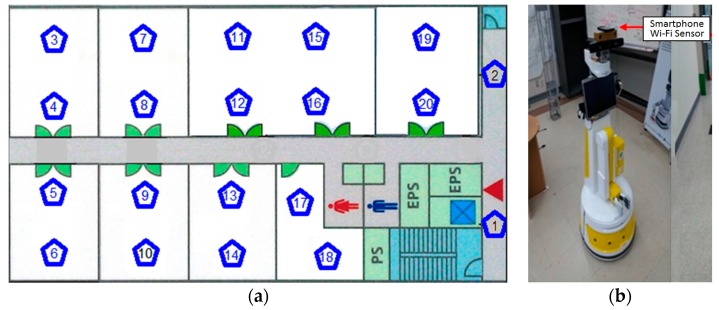
(**a**) The initial experiment floor map area with marked calibration locations. Later, the number of calibration locations are increased and decreased according to its resolution; (**b**) The robot attached with a smartphone Wi-Fi sensor used in sensing invariant Wi-Fi RSS data collection. The collected data are sent through the network and stored in a server.

The experiment is set up in a 400 m^2^ area on a floor of a building. There are 25 Wi-Fi signals available for fingerprinting with 20 calibration locations assigned based on 4 m × 4 m as the initial resolution. The resolution will be increased and decreased during the comparative investigation of different calibration location resolutions (resolution between 1 m × 1 m and 6 m × 6 m). The calibration locations are the point that has been used to collect the readings of the Wi-Fi RSS data. Figure 7a depicts the floor map of the experimental area with all 20 calibration locations (Loc. 1 to Loc. 20). Each calibration location is within equal distance from each other based on the specified resolution and it is being marked on the floor for easy identification. In the training phase, 100 invariant RSS data are collected at each calibration location to form invariant reference class patterns. To collect invariant RSS data with minimal random spatiotemporal disturbances, the data are collected after midnight when no random disturbance such as human is present. Furthermore, a smartphone is placed on a pinnacle of the head of a remotely controlled robot to avoid any blocking effect of the Wi-Fi sensor. The smartphone collects Wi-Fi RSS at a particular calibration location and the RSS data is sent to a server, and the readings are tagged to the location associated with it. Figure 7b illustrates the robot that has been used during data collection. However, during the testing stage in the localization phase, the smartphone could either be held by a human or attached to a robot to obtain a real spontaneous RSS measurement. Meanwhile, for performance comparison, the conventional non-invariant reference class patterns are formed with 100 spontaneous RSS data collected at each calibration location in normal office hours when people may be present in rooms and corridors. A Samsung Galaxy S4 smartphone with Android version 4.4.2 is used in the experiment.

### 4.2. Comparison of Success Rate and Its Temporal Variation

The first experiment is to compare the proposed method with the well-known conventional methods: *RADAR* [10] and *Removal of Useless Wi-Fi Sources* [11], in terms of the success rate at each calibration location and the possible variation of the success rate in time. The latter aims at investigating whether there is any degradation of performance with respect to the lapse of time, dictating recalibration. Success rate here is defined as the percentage of the total number of counts with correct recognition decision during testing divided by the number of total tests. The correct recognition decision is the precisely detected location as predicted by the localization system. To this end, the experiment has been carried out over the period of 18 weeks, where, each week, 100 localization experiments are conducted for four different orientations at each calibration location. Note that, for the sake of fair comparison, the same reference calibration locations of 4 m × 4 m resolution, as well as the same normal office hour environments, are adopted in the experiments.

**Figure 8:**
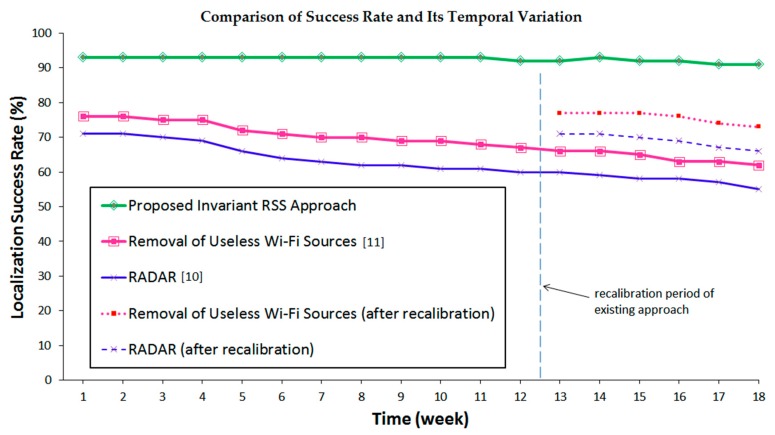
The success rate of the proposed method (green) in comparison with that of the conventional methods (red and blue), where the temporal variation over 18 weeks span is clearly shown. It is verified that the recalibration applied to the conventional methods puts their success rate back to the initial one.

The result based on 18 weeks of comparative testing is illustrated in Figure 8. The figure shows that the proposed invariant RSS-based method performs superior to conventional methods from the start of testing with the 93% success rate in comparison to the respective 76% and 71% by *RADAR* [10] and *Removal of Useless Wi-Fi Sources* [11]. The superior performance of the proposed method becomes even more evident as testing continues. For instance, after 18 weeks, the performance of conventional methods is degraded by 14% and 16% respectively, while that of the proposed method remains virtually constant. Note that, in between the week of 12 and 13, the reference pattern classes for the conventional methods are recalibrated to see how much it improves their success rate. It is found that the recalibration enables their success rate back to 77% and 71% respectively, similar to that of their first week performance, as expected. After the recalibration, it is also observed that the conventional methods return back to a pattern of performance deterioration.

### 4.3. Comparison of Success Rate at Different Resolution of Calibration Locations

**Figure 9:**
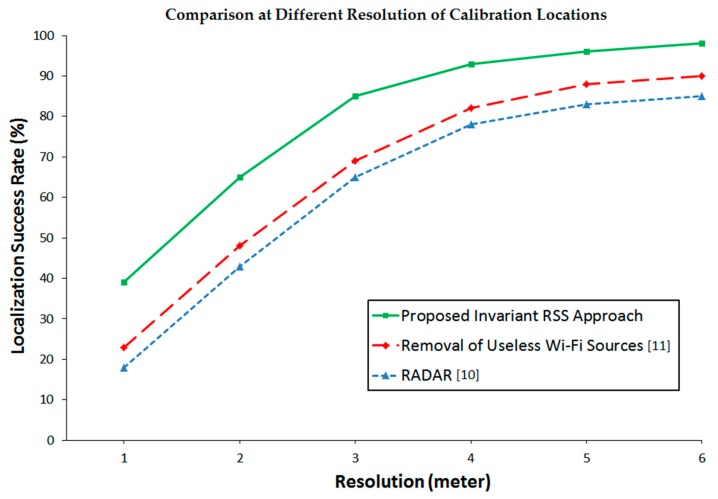
A comparative evaluation of the possible resolution of fingerprinting, where the proposed method (green) is able to achieve 90% success rate at 3.7-m resolution while the conventional methods (red and blue) fail even at 6-m resolution.

To have a comparative evaluation on the possible resolution of fingerprinting, the success rate of the proposed method at six different resolutions of calibration locations, ranging from 1 m to 6 m with 1 m interval, is compared with that of the conventional methods. The number of the calibration locations are increased and decreased when the resolution is higher and lower, respectively. The result is shown in Figure 9, where the proposed method is shown to achieve 90% success rate at 3.7-m resolution while the conventional methods fail to achieve 90% success rate even at 6-m resolution.

### 4.4. Comparison of Performance from Samples Collected over Different Length of Time

In the preceding experiments, the number of samples used for the formation of invariant reference pattern classes of our proposed method is *n* = 100 for each calibration location, which was taken over a period of two hours for all calibration locations of 4 m × 4 m resolution. The same number of samples is used for existing methods (*RADAR* and *Remove Useless APs*) for the formation of their location fingerprints. Our next investigation is to apply an increased number of samples from a longer period of time with the same resolution, which will be applied to all three approaches for performance comparison. The goal of this investigation is to check whether time length of collecting samples in the formation of reference pattern classes affecting the success rate.

We continue our investigation by increasing the number of samples *n* of each calibration location to *n* = 200 which was dispersedly collected in a one week time period. Then, we re-apply our approach by defining the invariant RSS statistics and forming the invariant reference pattern classes. To evaluate the performance comparison, we also apply the same number of samples from the same longer period of time in the formation of location fingerprints of existing approaches. We then repeat the same procedure for *n* = 300 which was collected in two weeks’ time period.

**Figure 10:**
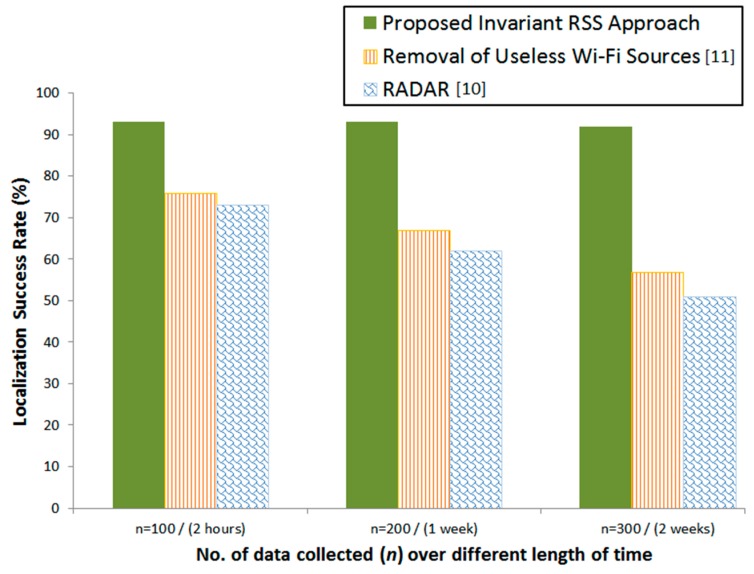
Performance comparison with a different number of samples collected over different time length. The result shows no degradation in our proposed method as the time length is increased in comparison to the conventional methods.

Figure 10 illustrates the comparison results. We observed that our invariant RSS approach produced the highest success rate from all tested approaches and it is stable regardless of the number of samples over a different length of time used. On the contrary, the conventional approaches suffer from success rate degradation as the number of samples increased over time. From our analysis, the reason behind this result is our approach only takes invariant RSS which maintain low variance although as time increased, as opposed to existing conventional approaches that take all available RSS where the variance is increased as the time is extended.

### 4.5. Computational Complexity Analysis

**Figure 11:**
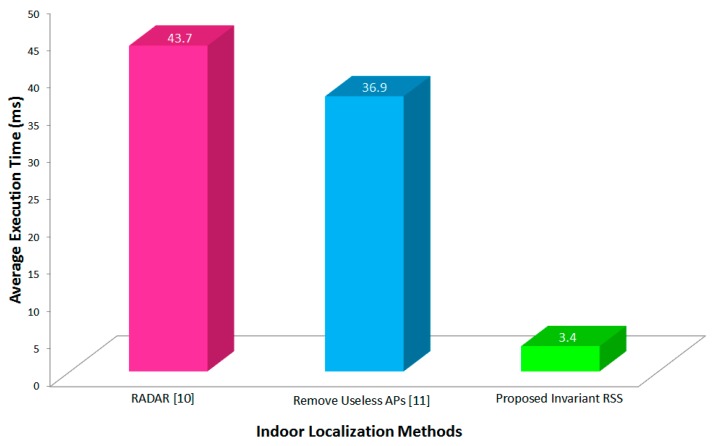
Comparison of average execution time for different methods: RADAR with α = 1, Remove Useless APs with α = 0.8, and Invariant RSS proposed with α = 0.3.

The computational complexity of Wi-Fi based fingerprinting is, in general, a function of the number of Wi-Fi sources *m* and of calibration locations *n*. Here, we represent the computational complexity as *O*(α*m* × *n*), α ≤ 1 with α representing the percentage of the Wi-Fi signals received that are selected to participate in classification. For instance, [11] excludes weak Wi-Fi signals from classification such that α becomes around 0.8. On the other hand, the proposed method excludes non-invariant Wi-Fi signals such that α becomes around 0.3, as shown in Figure 11. Note that, recent work by Sánchez-Rodríguez et al. [27] shows that a computational complexity of *O*(1) is feasible in terms of floating point computation based on multiple weighted decision trees although the decision trees may grow with O(α*m* × *n*).

### 4.6. Application Model Implementation

**Figure 12:**
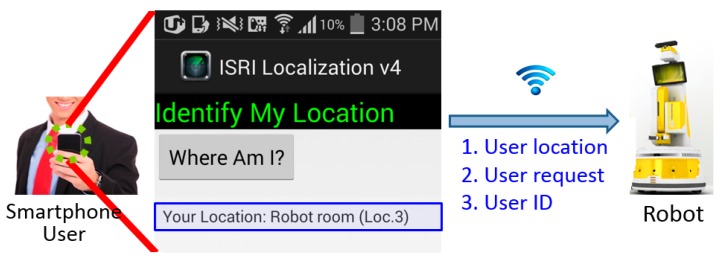
Smartphone’s indoor localization application information flow for initial human-robot interaction.

An actual implementation of the proposed method has been employed to localized human in a human-robot interaction case. Figure 12 illustrates the information flow of the smartphone application based on the proposed method’s algorithm for initial human-robot interaction. The smartphone user makes a location query through the application, the application will then capture spontaneous RSSs through the Wi-Fi sensor, check for effective invariant RSS, and then estimate the location. The user estimated location, the user request to the robot (if any), and the user identification will then sent to a robot for further action.

## 5. Theoretical Analysis

**Figure 13:**
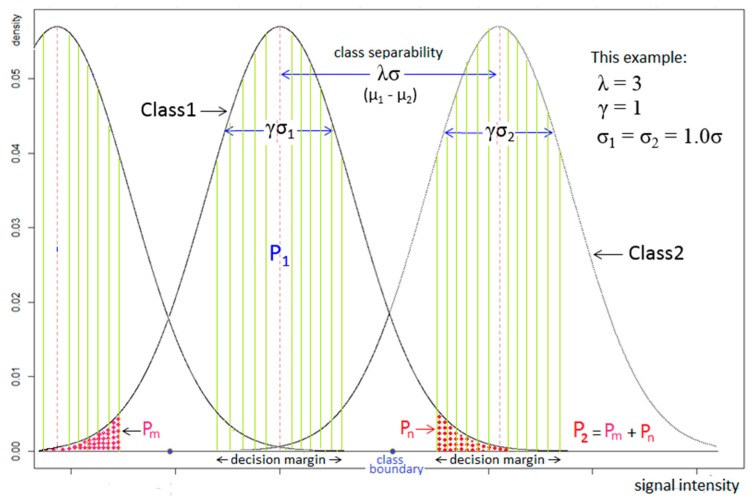
Schematic representation of different class distributions enlightening class separability, decision margins, correct decision probability, erroneous decision probability, and class boundary.

To understand the theoretical implication of the proposed method, a simulation study is conducted. To this end, the spontaneous RSS distributions at individual calibration locations are simulated for each Wi-Fi source same as a Gaussian distribution with their variances same as σ. Furthermore, the means of a pair of spontaneous RSS distributions are assumed separated by λσ, λ > 0, as illustrated in Figure 13 with the class separability of $\mu_{1}-\mu_{2}=3.0\sigma$.

Meanwhile, the invariant RSS distributions at individual calibration locations are modeled as having their means same as those of corresponding spontaneous RSS distributions but their variances represented indirectly by the decision margin, γσ (Note: γσ = ξσ(***s**_i,j_*) in Algorithm 1), γ < λ, around their means, as shown in Figure 13. Note that the above simulation model used for RSS distributions is not realistic but intended to show the performance of proposed method under harsh conditions. However, in our experiments, the sensor noise ***δ**_i,j_* is found to be bounded by 3 dBm independent of calibration locations. Therefore, the decision margin ξσ(***s**_i,j_*) in Algorithm 1 or γσ in Figure 13 is set as 3 dBm in real experiments.

Recall that, in our proposed method, the location query returns the calibration location *q*, if *q* satisfies:

(9) $$q=\underset{j}{\mathrm{Max}}\{\mathrm{Cardinal}\left[s_{i,j}\left(t\right)\in \xi \sigma \left(s_{i,j}\right),i=1,\ldots ,m\right],\;j=1,\ldots ,\;n\}$$

**Figure 14:**
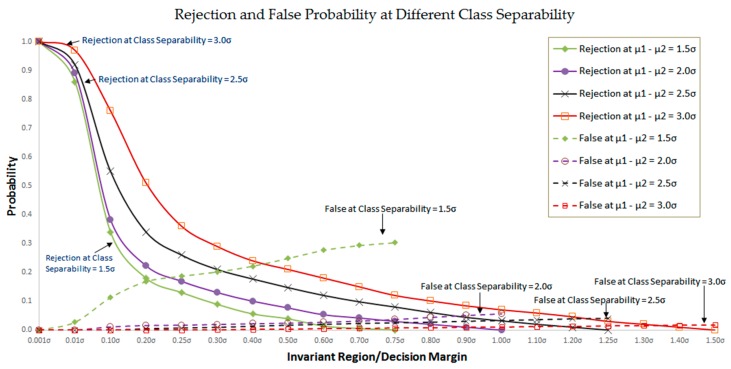
Rejection and False probability comparison for class separability between 1.5σ and 3.0σ.

The location query, Equation (9), is simulated by applying Bernoulli trial to the above RSS model as follows: First, let the probabilities of a Wi-Fi source fall within the decision margins of the respective right and wrong calibration locations be P_1_ and P_2_ (refer to Figure 13). Then, the probability that *l* and *m* Wi-Fi sources fall within the right and wrong calibration locations, respectively, can be computed by:

(10) $$\underset{m}{\sum }\left(\begin{array}{c} n\\ l+m \end{array}\right){\left(P_{1}+P_{2}\right)}^{l+m}\;{\left(1-P_{1}-P_{2}\right)}^{n-l-m}\left(\begin{array}{c} l+m\\ l \end{array}\right)Q^{l}{\left(1-Q\right)}^{m}$$

where $Q=P_{1}/\left(P_{1}+P_{2}\right)$. Note that *l* < *m*, *l* > *m*, and *l* = *m* implies respectively a false, a success, and a rejection in recognition. Figure 14 illustrates the false and rejection probabilities in terms of various decision margins and class separabilities. False probability is defined as the probability of the wrong detection is made, while rejection probability is defined as the probability of no decision is made because there is not enough information to make a decision. The figure shows that, given a class separability, the minimal false rate required can be achieved with a proper choice of decision margin, although the rejection rate goes up. The increased rejection rate can be handled by a suitable number of query repetitions. In practice, it suffices at most several query repetitions to reach a minimal false rate of decision.

**Figure 15:**
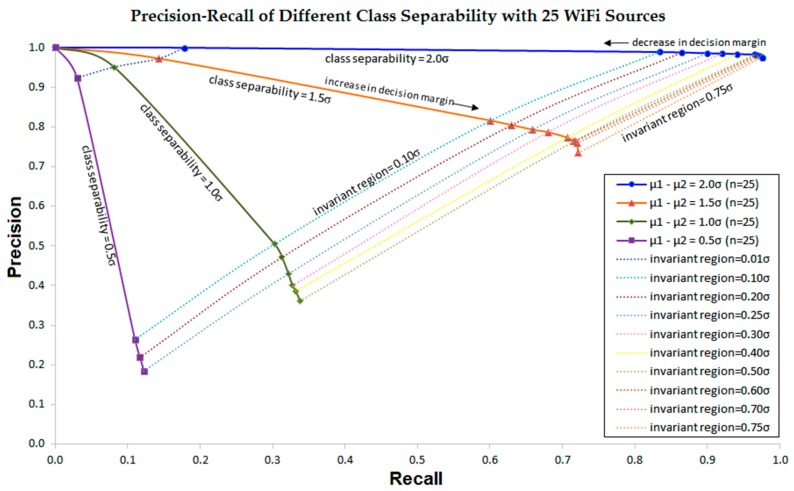
Precision-Recall curve of the proposed approach that shows the classifier performance with 25 Wi-Fi sources.

Next, Figure 15 depicts the precision-recall curve to show the effect of decision margin, representing the invariant region in our proposed method, on various class separabilities for the number of Wi-Fi sources given as 25. It represents how decreasing decision margin from the class boundary, the end of each precision-recall line, heightens the precision while lowering the recall. With the invariant region that decreases decision margin, the proposed classifier heightens the precision so that false positives seldom occur. Note that, in our classifier, resulting a decrease in recall increases rejections instead of increasing false negatives, such that repeating location queries ultimately produces true positives.

**Figure 16:**
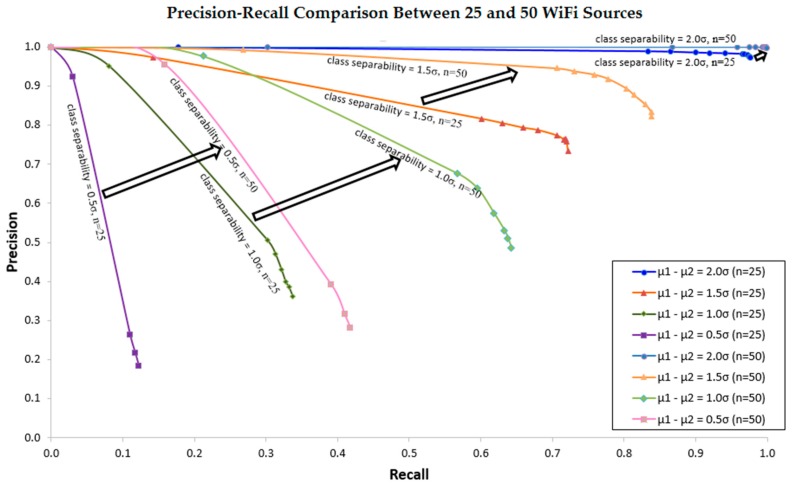
Precision-Recall curve changes due to increasing in the number of Wi-Fi sources from 25 to 50. The arrows demonstrate the respective changes from 25 to 50 Wi-Fi sources at different class separability.

Figure 16 shows how the precision-recall curve changes due to the increase in the number of Wi-Fi sources from 25 to 50. It clearly shows the dramatic improvement of the precision-recall curve with the increase in the number of Wi-Fi sources. Figure 15 and Figure 16 also indicates that, by properly choosing decision margin or invariant region and the number of Wi-Fi sources, we can set the precision and the recall or the rejection rate optimal for the given class separabilities.

## 6. Conclusions

As demonstrated by the experiment, the proposed method yields more than 17% higher success rate and more than 40% improvement in the resolution of fingerprinting (3-m vs. 5-m at 85% success rate), compared to conventional methods [10,11]. Furthermore, the proposed method provides solid stability of performance in time, contrary to conventional methods experiencing performance degradation in time. Besides the better performance in success rate, accuracy, and stability, the proposed method also has lower execution time, where the average execution time is more than 10 times faster than the conventional methods. The proposed method is expected to find many smartphone-based indoor location fingerprinting applications.

## Figures and Tables

**Table 1:** Setup Parameter of the Invariant Reference RSS Generation.

No. of Wi-Fi Signal Sources	No. of Calibration Locations
20	7
50	
80	
20	15
50	
80	
20	20
50	
80	
